# The evolving landscape of primary headache therapy

**DOI:** 10.1016/j.neurot.2026.e00931

**Published:** 2026-05-23

**Authors:** P.R. Holland, P.J. Goadsby

**Affiliations:** a Headache Group, Wolfson Sensory Pain and Regeneration Centre, Institute of Psychiatry, Psychology and Neuroscience, King’s College London, London, UK; b Division of Biomedical Sciences, King Abdullah University of Science and Technology, Thuwal, Saudi Arabia

**Keywords:** Migraine, Headache, Therapy, Advances

Migraine serves as a success story in translational neurology, with an increased understanding of disease mechanisms [1] and recent therapeutic advances for both acute and preventive treatment [2,3]. Despite this success, migraine remains one of the leading causes of disability globally [4], and with the recent discontinuation of lasmiditan [5], a first in class serotonin 5-HT_1F_ receptor agonist [6,7], the therapeutic tool kit, especially for patients with cardiovascular risk, has taken a step in the wrong direction.

With this in mind, Gamel-Eltrabily, Salinas-Abarca, Romero-Reyes, and Akerman discuss the therapeutic pipeline in migraine in this issue of Neurotherapeutics, discussing several novel targets under development [8]. The review highlights the emerging role of targeting specific ion channels, including ATP-sensitive potassium channels (K_ATP_), large conductance calcium- and voltage-activated potassium channels (BK_Ca_), and transient receptor potential melastatin (TRPM) channels. Importantly, blockade of one of these targets, TRPM8 has successfully completed a phase-2b trial [9] strengthening the continued translation from bench to bedside in migraine, despite a prior lack of efficacy for TRPM3 blockade, reported as part of a U.S. Securities and Exchange Commission filing [10]. Further, building on the success of targeting neuropeptides in migraine, the authors discuss recent updates on targeting hypothalamic-related peptides, including oxytocin, orexin and prolactin. The hypothalamus has emerged as a key regulator of migraine [11], forming part of a complex network alongside brainstem structures that likely influence attack susceptibility thresholds.

In parallel to the focus on hypothalamic-related neuropeptides; Holland and Fronczek discuss the potential for chronotherapy in migraine [12]. Approximately half of all people with migraine report a rhythmic circadian pattern of attacks [13], governed by the master biological clock, located in the hypothalamic suprachiasmatic nucleus. Critically, people with migraine are more likely to have extreme chronotypes [14] (genetic tendency to sleep at times that are significantly earlier or later than the average population) and disruption of normal circadian rhythms increases migraine attack susceptibility and attack frequency [13,15,16]. Therefore, harnessing the positive effects of targeting biological rhythms, particularly circadian rhythms, offers a novel personalized approach to reduce migraine attack susceptibility and align interventions with an individual’s natural 24-h cycle.

Continuing with the focus on personalized medicine, sex-specific mechanisms are being increasingly integrated into migraine therapy in an attempt to improve treatment outcomes. Herein, Rubio-Beltran and Maassenvandenbrink review sex-specific considerations in migraine [17]. Migraine is predominantly a female disorder, with up to 3 times more migraine in women than men [18], the potential mechanisms of which are discussed, including the effect of sex-hormones. Despite this female preponderance, clinical trial data has not demonstrated convincing sex-specific differences in response to the triptans: serotonin 5-HT_1B/1D_ receptor agonists, albeit with differential adverse event profiles [19], nor consistently to calcitonin gene-related peptide (CGRP) targeted therapies [20,21]. This issue and other sex-specific responses to therapeutic interventions are discussed across the female life-cycle, identifying major knowledge gaps and promoting the integration of sex- and gender-stratified approaches to facilitate improved personalized migraine care.

Non-neuronal targets in migraine, particularly glia and other immune cells, represent developing research themes due to their roles in neuroinflammation and sensitization. In the first of two reviews on non-neuronal targets, Gligam Feliu-Soler and Vila-Pueyo discuss the role of non-neuronal targets for migraine therapy [22]. Key targets include microglia, astrocytes, satellite glial cells, mast cells, Schwann cells, and macrophages, which when activated likely influence migraine-related pain and cortical spreading depression, the underlying mechanism of migraine aura [23]. Many of these non-neuronal targets respond to established neuropeptides, e.g. calcitonin gene-related peptide, (CGRP)) [3] and emerging neuropeptides, e.g. pituitary adenylate cyclase activating peptide, (PACAP) [24,25], that are considered to play a central role in migraine pathogenesis. Stemming from this, in the case of mast cells, protease-activated receptor 2 has emerged as a novel translational target, currently under phase 2 clinical trial [26]. In the second review, Nematgorgani, Brandon, and Dussor continue the focus on mast cell targets, reviewing the potential role of the mast cell specific receptor Mas-related G protein-coupled receptor X2/B2 (MRGPRX2/B2) as an important contributor to neuroimmune interactions in migraine [27]. Both CGRP and PACAP can induce mast cell degranulation via MRGPRX2/B2, leading to neuronal sensitization and, thus, blockade of MRGPRX2/B2 is considered a novel target for new migraine therapeutics.

Finally, Della Pietra and Russo, review the potential of targeting the endocannabinoid system for migraine [28]. Offering analgesic, anti-inflammatory and neuroimmune regulatory properties, the review discusses the overlap between endocannabinoid signalling and migraine pathophysiology, focusing mainly on anandamide and 2-arachidonoylglycerol pathways. While, clinical translational data are limited, the authors highlight the potential for future targeted modulation of the endocannabinoid system, acting via diverse mechanisms including modulation of circadian rhythms [12] and brain glymphatic clearance [29], two emerging mechanisms, the dysfunction of which is considered to increase migraine susceptibility.

The advances covered in this special issue offer encouragement that the translational pipeline of novel migraine therapeutics continues at pace. Each review offers mechanistic insight, clinical implications and where appropriate practical considerations that together describe a path from discovery science to clinical impact.

## Declaration of competing interest

The authors declare the following financial interests/personal relationships which may be considered as potential competing interests
